# Freeze-Drying of Pharmaceuticals in Vials Nested in a Rack System—Part I: Freezing Behaviour

**DOI:** 10.3390/pharmaceutics15020635

**Published:** 2023-02-14

**Authors:** Roberto Pisano, Fiora Artusio, Marco Adami, Antonello A. Barresi, Davide Fissore, Maria Chiara Frare, Francesco Zanetti, Gabriele Zunino

**Affiliations:** 1 Department of Applied Science and Technology, Politecnico di Torino, 24 Corso Duca degli Abruzzi, 10129 Torino, Italy; 2 Independent Consultant, 20100 Milano, Italy; 3 Stevanato Group, 17 Via Molinella, 35017 Piombino Dese, Italy

**Keywords:** freezing, cryopreservation, freeze-drying, rack system, tubing vials, cooling rate, heat transfer, proteins, sucrose, lactate dehydrogenase

## Abstract

The distribution of biopharmaceuticals often requires either ultra-cold conditions or lyophilisation. In both cases, the drug product is frozen and, thus, exposed to similar stress conditions, which can be detrimental to its quality. However, these stresses can be inhibited or mitigated by a suitable formulation and/or an appropriate freezing design. This paper addresses how the key freezing parameters, i.e., ice nucleation temperature and cooling rate, impact the freezing behaviour of a sucrose-based formulation. The analysis included two loading configurations, vials directly resting on the shelf and nested in a rack system. The loading configuration affected the product freezing rate and the ice nucleation temperature distribution, resulting in larger ice crystals in the case of vials nested in a rack system. SEM micrographs and specific surface area measurements confirmed the different product morphology. Eventually, the different product morphology impacted the bioactivity recovery of lactate dehydrogenase.

## 1. Introduction

The distribution and storage of almost all biopharmaceuticals require either cold, i.e., 2–8 °C, or ultra-cold conditions, i.e., −20 or −70 °C. However, a cold-chain system is prohibitively costly, e.g., approximately 80% of the cost of vaccination programs in developing countries comes from low-temperature storage [1]. Furthermore, any failure in cold-chain integrity can result in significant losses of drug products. For example, in 2011, the World Health Organisation (WHO) estimated that 2.8 million vaccine doses were lost because of cold-chain failures [2].

As the cold chain remains an inefficient distribution system, alternative routes of formulating actives are used to stabilise biopharmaceuticals at higher temperatures. For example, many drug products are currently commercialised as lyophilised powders, which are stable at room temperature or under moderate refrigeration conditions. Maintaining a chilled chain is not less challenging than a chain for frozen commodities, and thus, most lyophilised products are ideally designed to be stable at room temperature. However, even in the case of lyophilised products, the drug formulation has to be frozen before drying, and this process can result in an undesired loss of its therapeutic potency [3]. For example, therapeutic proteins can denature at low temperatures because of the reduced penalty for water–hydrophobic interactions [4,5,6]. Additionally, the formation of a new solid–liquid interface, i.e., the ice–water interface, can result in a marked loosening of the native protein fold [7,8,9], and the progressive separation of water can result in a rapid change in ionic strength and amorphous phase composition, potentially enhancing the rate of chemical reactions. Eventually, pH shifts, the phase separation of polymers, and the selective crystallisation of some excipients can impair protein activity [10]. Overall, a large variety of phenomena can harm protein stability, some more stressful than others.

Generally, forming large ice crystals benefits protein stability [11,12,13] as it reduces the extension of the ice–water interface. To this purpose, two parameters can be manipulated to control the average size of ice crystals: the cooling rate [14] and the nucleation temperature [15]. More specifically, a high cooling rate, e.g., by immersion in liquid nitrogen, promotes the formation of small ice crystals [16,17]. Nevertheless, in the case of freeze-drying, a narrow range of cooling rates can be implemented in an industrial unit, i.e., from 0.1 to 1 °C min^−1^, and, thus, the ice crystals’ size is predominantly determined by the ice nucleation temperature; high nucleation temperatures promote the formation of large ice crystals [17,18]. Unfortunately, the nucleation temperature is stochastically distributed and hard to control precisely [19]. For example, Pisano and Capozzi [19] observed that ice nucleation temperature within a batch of vials is widely distributed between −5 and −25 °C. Recently, new technologies have been proposed for triggering ice nucleation within a narrow range of temperatures [20,21], but their implementation in manufacturing units is still under investigation [21,22].

The stochasticity of ice nucleation inevitably leads to variations in the freezing behaviour among vials of the same batch, even if they are subjected to the same thermal environment. Consequently, vial-to-vial variations are commonly observed in product morphology, residual biological activity, and, in the case of lyophilisation, residual moisture [23]. Since these variations may affect the critical quality attributes of the frozen and lyophilised drug products, they should be considered in the design of the freezing process. In this perspective, many authors have recently used mathematical modelling to predict the ice nucleation temperature distribution within a batch of vials nested in pallets [24,25] and have correlated this distribution to the frozen product morphology [26,27,28]. Deck et al. [25] also observed that batch heterogeneity results from the interplay between stochastic ice nucleation and the thermal interaction between neighbouring vials. It was hypothesised that the heat released by a nucleated vial could potentially delay the ice nucleation in the surrounding vials and, hence, modify the induction time distribution. Of course, this phenomenon will be more or less accentuated depending on the heat transfer efficiency between adjacent vials, which, in turn, depends on the loading configuration.

Various authors have investigated the influence on batch uniformity of packing density and loading configuration, as well as the role of neighbouring vials and their holders made of different materials, but all these works have focused on heat transfer during primary drying [29,30,31,32]. Among others, Ehlers et al. [32] studied the impact of neighbouring vials and the influence of vial separation and distance during primary drying, but they argued that the freezing process might also be influenced.

In this paper, we have investigated how the stochastic distributions of the ice nucleation temperature change with the loading configuration of the vials. Two configurations will be specifically addressed; vials directly resting on the temperature-controlled shelves and vials nested in a rack system. This last configuration derives from the fact that some pharmaceutical companies and contract manufacturing industries conduct the aseptic handling and filling process using the vials as they are distributed in sterile form by the glass containers’ manufacturer, including its plastic sterilised secondary packaging in the form of a rack system. This practice allows for the simplification of the vial handling operation in a sterile pharmaceutical manufacturing environment. In this configuration, the vials are physically separated by the rack system housing; thus, the thermal interaction between adjacent vials should be mitigated, potentially influencing the nucleation time distribution.

Furthermore, the vials nested in a rack system are not in direct contact with the temperature-controlled shelf. Therefore, we expect that the equipment-to-vial heat transfer resistance differs from that observed for the conventional loading configuration, wherein all the vials are in direct contact with the equipment shelves. The second part of this study will address the influence of nesting vials in a rack system on the heat transfer efficiency and batch heterogeneity during primary drying and compare this loading configuration with the case of vials resting on the shelf.

## 2. Materials and Methods

### 2.1. Materials

All the freezing experiments were conducted using a 5 wt% sucrose solution in 4 cc tubing vials (2R ISO, Stevanato Group, Piombino Dese, Italy). Sucrose was purchased from Merck and used without further purification, while the solutions were prepared using water for injection (Fresenius Kabi, Milan, Italy). All the reagents are of analytical grade.

**Figure 1:**
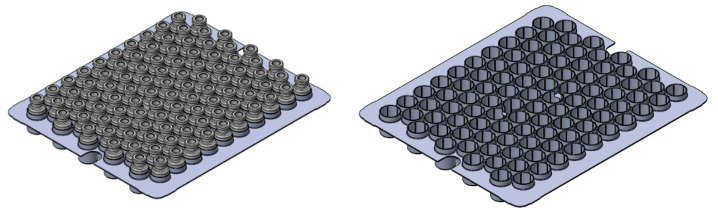
Schematic of the rack system provided by Stevanato Group (**left**) with and (**right**) without vials.

In total, 100 tubing vials were inserted into dedicated alveolar secondary packaging (SG EZ-Fill^®^ Nest, Stevanato Group, Piombino Dese, Italy). As shown in Figure 1, tubing vials in this rack system, also named nest, were in a hexagonal arrangement and raised approximately 1 mm above the shelf. An arrangement of this kind enabled the vials to not be in direct contact with one another or the shelf.

### 2.2. Experimental Set-Up

The freezing runs were carried out in a lab-scale freeze-dryer (Revo, Millrock Technology, Kingston, NY, USA) using two loading configurations; vials were directly loaded on the temperature-controlled shelf and nested in a rack system. In both configurations, two video cameras were placed on opposite sides of the shelf to monitor the freezing behaviour of approximately 100 vials at a frame rate of 5 fps.

For each monitored vial, the ice nucleation time ($t_{n}$) corresponded to the instant in which opacity increased in the liquid. More specifically, $t_{n}$ refers to the time elapsed since the temperature of a reference vial, detected through a T-type miniature thermocouple (Tersid, Milan, Italy) placed in close contact with the vial bottom, reaches 0 °C. The analysis was limited to central vials as the edge-vial effect could bias the results. Similarly, the reference vials were excluded from the analysis as the thermocouple tips can perturb the ice nucleation event.

All the vials were filled with 2 mL of a 5 wt% sucrose solution, preliminarily filtered through a 0.22 µm syringe filter (PVDF, Merck, Milan, Italy). The filling operation was carried out within a laminar hood to limit dust contamination, and immediately after filling, vials were loaded into the freezing chamber at room temperature.

Samples were frozen at a cooling rate of 0.25 °C min^−1^ and held at −45 °C for 30 min. Then, they were thawed at a heating rate of 3 °C min^−1^ and held at +30 °C for 60 min to promote their complete melting. Furthermore, as the cooling rate could influence the ice nucleation time distribution, two further runs were conducted, varying the cooling rate, i.e., 0.5 and 1 °C min^−1^. These freeze–thawing runs were carried out in triplicate.

### 2.3. Determination of the Ice Nucleation Time Distribution

Two cameras monitored the freezing behaviour of approximately 100 vials and detected the induction time for ice nucleation as the instant in which the solution became opaque. For all the freezing runs, the induction time was always in the range of 0 to 125 min; hence, the cumulative nucleation time distribution $\left(F_{n,i}\right)$: was determined by referring to 5-minute-width classes:

(1) $$F_{n,i}\left(t_{n,i}\right)=\frac{\sum_{j=1}^{i}N_{j}\left(t_{n,j}\right)}{N_{t}}$$

where $F_{n,i}$ is the cumulative fraction of nucleated vials belonging to the i class, $N_{j}$ is the number of vials belonging to the j class, $N_{t}$ is the total number of monitored vials, and $t_{n,j}$ is the nucleation time of the j vials. The time at which the cumulative distribution is 0.5 (*t*_50_) is defined as the median nucleation time, while the difference between the time at which it is 0.1 (*t*_10_) and 0.9 (*t*_90_) defines its width. Eventually, the interquartile range of the induction time distributions (*t*_IQR_) gives a quantitative estimation of the statistical dispersion of the data and is defined as the difference between the 75th and 25th percentiles.

If we assume that all the vials have the same temperature as that of the reference one, the nucleation time $t_{j}$ can be translated into a nucleation temperature $T_{n,j}$, and hence, the cumulative distribution of the ice nucleation temperature is:

(2) $$F_{n,i}\left(T_{n,i}\right)=\frac{\sum_{j=1}^{i}N_{j}\left(T_{n,j}\right)}{N_{t}}$$

where $F_{n,i}$ is the cumulative fraction of nucleated vials belonging to the *i* class, $N_{j}$ is the number of vials belonging to the *j*-class, $N_{t}$ is the total number of monitored vials, and $T_{n}$ is the ice nucleation temperature. The temperature at which the cumulative distribution is 0.5 (*T*_50_) is defined as the median nucleation temperature, while the difference between the temperature at which it is 0.1 (*T*_10_) and 0.9 (*T*_90_) defines its width. Eventually, the interquartile range of the temperature distributions (*T*_IQR_) gives a quantitative estimation of the statistical dispersion of the nucleation temperature data.

### 2.4. Determination of the Overall Equipment-to-Vial Heat Transfer Coefficient 

As the liquid is cooled, the heat flow rate ($j_{q}$) between the shelf and the vial bottom, using a simplified and mono-dimensional approach, can be described as:

(3) $$j_{q}=UA\left(T_{s}-T_{\ell }\right)$$

where U is the overall heat transfer coefficient in W m^−2^K^−1^, $T_{s}$ is the shelf temperature in K, $T_{\ell }$ is the liquid temperature in K, and A is the heat transfer area in m^2^. In this study, this area was conventionally defined, for both loading configurations, as the cross-sectional area of the vial, while the heat transfer driving force is defined as the difference between the shelf temperature and that of the solution at the vial bottom. If the temperature evolution of the liquid being frozen is measured, $j_{q}$ can also be estimated as:

(4) $$j_{q}=\rho_{\ell }V_{\ell }{\hat{c}}_{p,\ell }\frac{dT_{\ell }}{dt}$$

where $\rho_{\ell }$, ${\hat{c}}_{p,\ell }$ and $V_{\ell }$ are respectively the mass density (in kg m^−3^), the specific heat capacity (in J kg^−1^K^−1^), and the volume (in m^3^) of the liquid being frozen. Combining Equations (4) and (5), the overall heat transfer coefficient between the shelf and the vial bottom is:

(5) $$U=\frac{1}{A\left(T_{s}-T_{\ell }\right)}\rho_{\ell }V_{\ell }{\hat{c}}_{p,\ell }\frac{dT_{\ell }}{dt}$$

where U is the effective heat transfer coefficient that results from the contribution of various heat transfer mechanisms, including the conduction through the vial wall and, in the case of the rack system, within the nesting fins. These aspects will be further investigated in the second part of this work, but this parameter is introduced here to roughly compare the heat transfer efficiency for the two loading configurations. Of course, in the case of vials nested in a rack system, the heat transfer mechanisms involved are more complex, and the vial-side wall contribution might be more relevant. For these reasons, U is here generically defined as the equipment-to-vial overall heat transfer coefficient.

### 2.5. Lyophilised Product Morphology Characterisation

The internal structure of the lyophilised cake was assessed using a scanning electron microscope (SEM, FEI type, Quanta Inspect 200, Eindhoven, the Netherlands). The samples were frozen at a cooling rate of 0.5 °C min^−1^ and held at −45 °C for 2 h; primary drying was carried out at −25 °C and 10 Pa for 63 h, followed by secondary drying (3 h) at the same pressure and 20 °C reached with a 4 h ramp (0.2 °C/min). The operating conditions guaranteed that the product temperature was below the glass transition temperature (−32 °C during primary drying) for the whole process [33]. For each sample, the SEM images were taken at its centre, and approximately 100 pores per image were selected and approximated to an ellipse. The pore size was estimated as the diameter of the circle having the same area-to-perimeter ratio of the approximating ellipse.

The specific surface area (SSA) of the prepared powders was calculated using the model of Brunauer, Emmett, and Teller (BET) using a Micromeritics ASAP 2020 (Micromeritics, Norcross, GA, USA) apparatus. Approximately 200 mg of lyophilised powder was loaded into the glass BET sample cell and then degassed at 40 °C for 3 h. The nitrogen adsorption–desorption isotherm was measured at −196 °C over a relative pressure (*P*/*P*_0_) range of 0.05–0.30.

### 2.6. Residual Biological Activity of Lactate Dehydrogenase

Lactate dehydrogenase (LDH) from rabbit muscle (Merck, Milan, Italy) was dialysed against 10 mM sodium citrate buffer (pH 6.5) at 5 °C. The buffer was changed three times: after 3 h for the first two dialysis cycles, whereas the third cycle lasted overnight. After dialysis, the LDH concentration was measured spectrophotometrically at 280 nm (Multiskan FC Microplate Photometer, Thermo Fisher Scientific, Milan, Italy) using an extinction coefficient of 1.44 mL/(mg cm). The aliquots, containing 200 µg/mL of LDH, were then stored at −80 °C.

Before testing, the LDH aliquots were thawed at room temperature and diluted in citrate buffer to 10 µg/mL. All the samples underwent two freeze–thawing runs. During freezing, samples were cooled to −45 °C at 0.25 °C min^−1^ and then thawed to +20 °C at 1 °C min^−1^. The LDH enzymatic activity was measured from the increase in the absorbance signal at 450 nm following the reduction of NAD to NADH at 37 °C after 30 min. A similar test was repeated at a higher cooling rate, i.e., 1 °C min^−1^.

## 3. Results

### 3.1. Vials in Direct Contact with the Shelf

**Figure 2:**
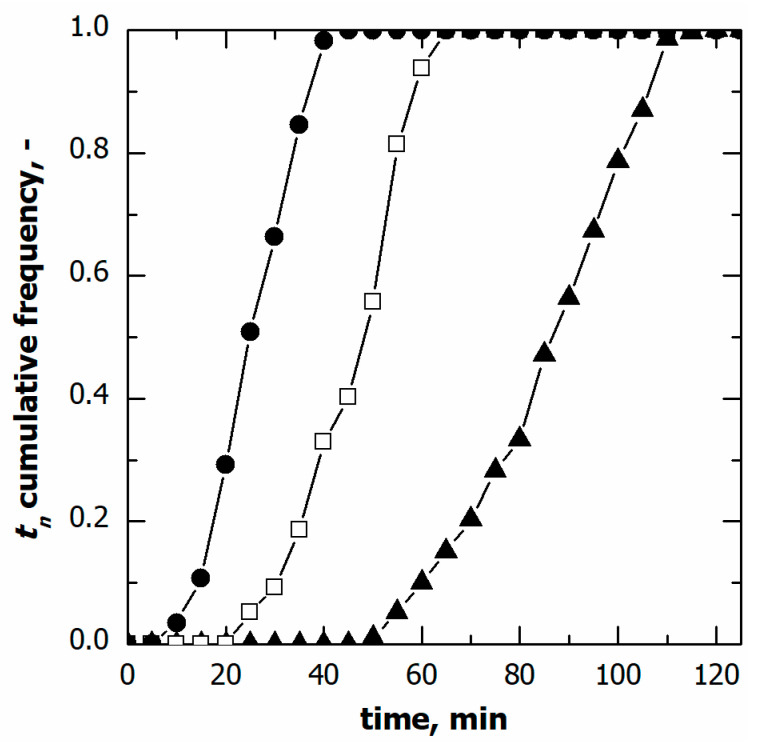
Cumulative distribution for the ice nucleation time in the case of vials in direct contact with the shelf. Data refer to various cooling rates: (▲) 0.25, (□) 0.50, and (●) 1.00 °C min^−1^.

Figure 2 shows the cumulative distributions of the ice nucleation time in the case of vials in direct contact with the shelf and three different cooling rates, while Table 1 gives the percentile values of the cumulative nucleation time and temperature distributions. The median nucleation time (*t*_50_) increased as the cooling rate decreased. Furthermore, the difference (*t*_90_-*t*_10_), which gives a quantitative estimation of the width of the cumulative distribution, decreased as the cooling rate increased. These results were expected since, for a given time, a higher cooling rate corresponds to higher supercooling and, therefore, a higher probability that nucleation occurs. It follows that nucleation took place, on average, in a shorter time as the cooling rate increased.

**Figure 3:**
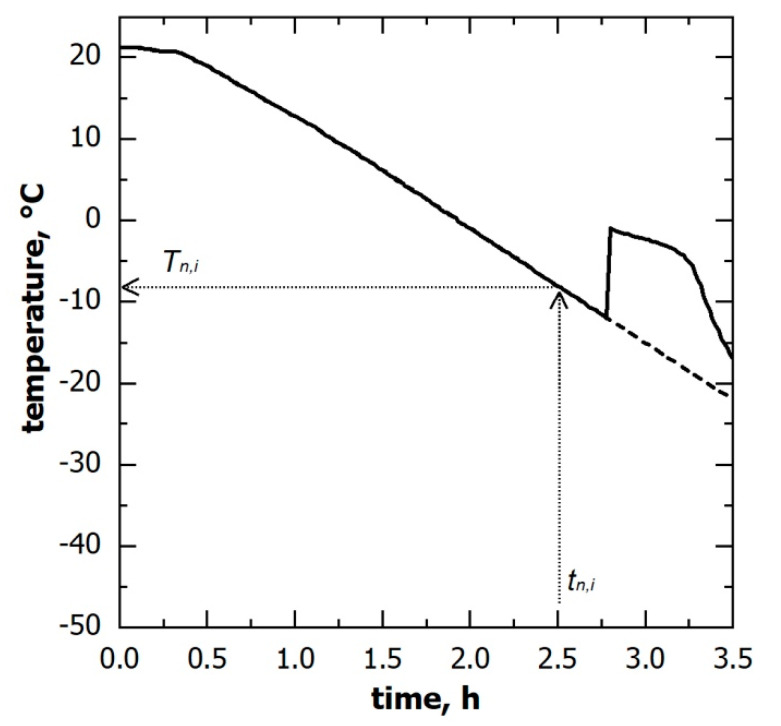
Temperature evolution of the solution being frozen, as observed through thermocouples; (solid line) the measured signal and (dashed line) the profile used to derive the nucleation temperature distribution. A corresponding nucleation temperature ($T_{n,i}$) can be identified for each ice nucleation time ($t_{n,i}$) observed in a non-monitored vial.

If the temperature of the supercooled liquid is known, a specific nucleation temperature ($T_{n,i}$) corresponds to a given nucleation time ($t_{n,i}$), as shown in Figure 3. Therefore, the nucleation temperature distribution can be derived from the corresponding nucleation time distribution. Here, the supercooled liquid temperature was measured through miniature thermocouples. However, the thermocouples’ reading can be considered representative of the batch as a whole only as long as the monitored vials do not nucleate. Once ice nucleation occurs, the monitored vial temperature rapidly increases and, thus, is no longer representative of those vials wherein ice nucleation has not yet occurred. To correct this bias, the thermal profile after nucleation was extrapolated considering the signal of the thermocouples before nucleation, as shown in Figure 3.

**Figure 4:**
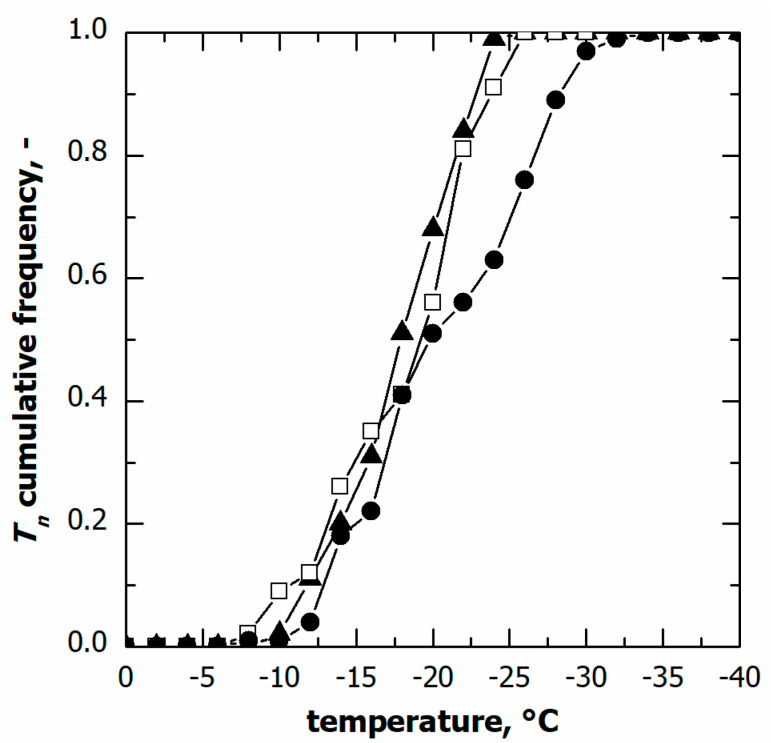
Cumulative distribution for the ice nucleation temperature in the case of vials in direct contact with the shelf. Data refer to various cooling rates: (▲) 0.25, (□) 0.50, and (●) 1.00 °C min^−1^.

The nucleation temperature distributions in the case of various cooling rates are compared in Figure 4. Although the nucleation time distributions varied with this parameter, the cumulative distribution of the nucleation temperature was independent of the cooling rate, at least in the range of 0.25 to 0.5 °C min^−1^. Within this range of conditions, these results suggest that the ice nucleation temperature distribution can be considered independent of the cooling rate used. 

On the contrary, at the highest cooling rate, i.e., 1 °C min^−1^, a significant fraction of vials (approximately 30%) nucleated below −25 °C, although the median nucleation temperature remained unvaried. This result was consistent with the spread of nucleation temperature data, $\left(T_{10}-T_{90}\right)$ and *T*_IQR_. Therefore, pronounced vial-to-vial variability in freezing behaviour and, hence, product morphology is expected in the case of a cooling rate higher than 0.5 °C min^−1^. These conditions are generally achievable in the case of samples loaded on a pre-cooled environment or cryogenically refrigerated freeze-dryers. 

### 3.2. Vials Nested in a Rack System

**Figure 5:**
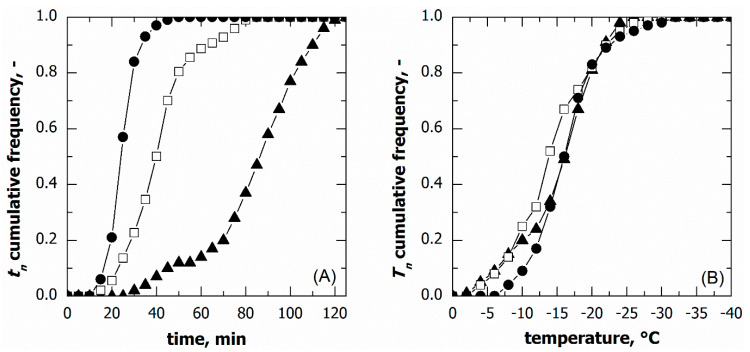
Cumulative distribution for (**A**) the ice nucleation time and (**B**) the ice nucleation temperature in the case of vials nested in a rack system. Data refer to various cooling rates: (▲) 0.25, (□) 0.50, and (●) 1.00 °C min^−1^.

The freezing behaviour of vials nested in a rack system was similar to that observed for the vials in direct contact with the shelf even if, as shown in Figure 5A and Table 1, the ice nucleation time distribution varied with the cooling rate. A higher cooling rate resulted in a narrower distribution and a shorter median nucleation time.

Nonetheless, the nucleation temperature distributions at various cooling rates were barely distinguishable, see Figure 5B. Therefore, even in the case of vials nested in a rack system, the nucleation temperature distribution did not depend on the cooling rate, at least in the range of conditions investigated.

### 3.3. Comparison between Vials in Direct Contact and Nested in a Rack System

**Figure 6:**
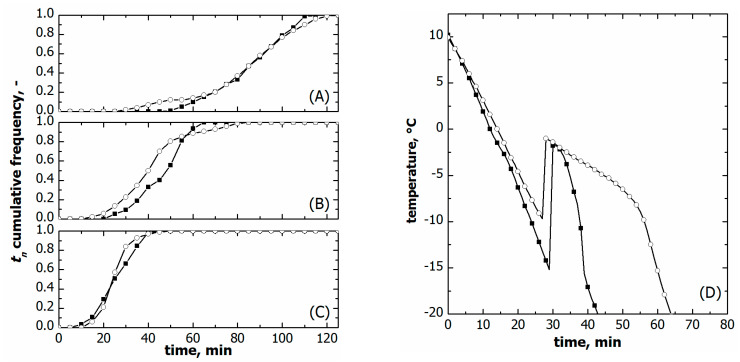
Comparison between cumulative ice nucleation time distributions of vials nested in a rack system (○) and in direct contact with the shelf (■). Data refer to various cooling rates: (**A**) 0.25, (**B**) 0.50, and (**C**) 1.00 °C min^−1^. (**D**) Comparison between the liquid temperature response during freezing in the case of vials nested in a rack system (○) and direct contact with the shelf (■). Data refer to a cooling rate of 1.00 °C min^−1^. The initial time was set as the instant at which thermocouples reached +10 °C.

Figure 6A–C compare the ice nucleation time distribution for the two loading configurations varying the cooling rate. 

The time distributions were comparable at the highest cooling rates, i.e., 1 °C min^−1^. On the contrary, *t*_10_ was systematically smaller for the vials nested in a rack system in the case of slow and moderate freezing, i.e., 0.25 and 0.5 °C min^−1^. Eventually, *t*_90_ did not change with the loading configuration at the same cooling rate. 

So as to better understand this behaviour, the temperature profiles of the supercooled liquid were compared for the two loading configurations on a constant cooling rate. As can be seen in Figure 6D, the temperature profile of the supercooled liquid varied with the loading configuration even if the same freezing conditions were used. This result can reasonably be attributed to a different heat transfer resistance between the vials and the equipment.

The heat transfer resistance is intuitively higher for the vials nested in a rack system as they are not in direct contact with cooling shelves. Figure 6D confirms this hypothesis; the vials nested in a rack system cooled more slowly than those directly resting on the shelf. The overall equipment-to-vial heat transfer coefficient (U) confirmed this qualitative behaviour. U was 48.7 ± 5.8 W m^−2^K^−1^ for the vials nested in a rack system and 77.5 ± 7.2 W m^−2^K^−1^ for those directly resting on the shelf.

Consequently, the ice nucleation time distributions of the two loading configurations cannot be directly compared because of variations in the reference time and actual freezing rate. Nevertheless, such a comparison is doable for the ice nucleation temperature distributions as they account for the specific liquid temperature response in the two loading configurations.

**Figure 7:**
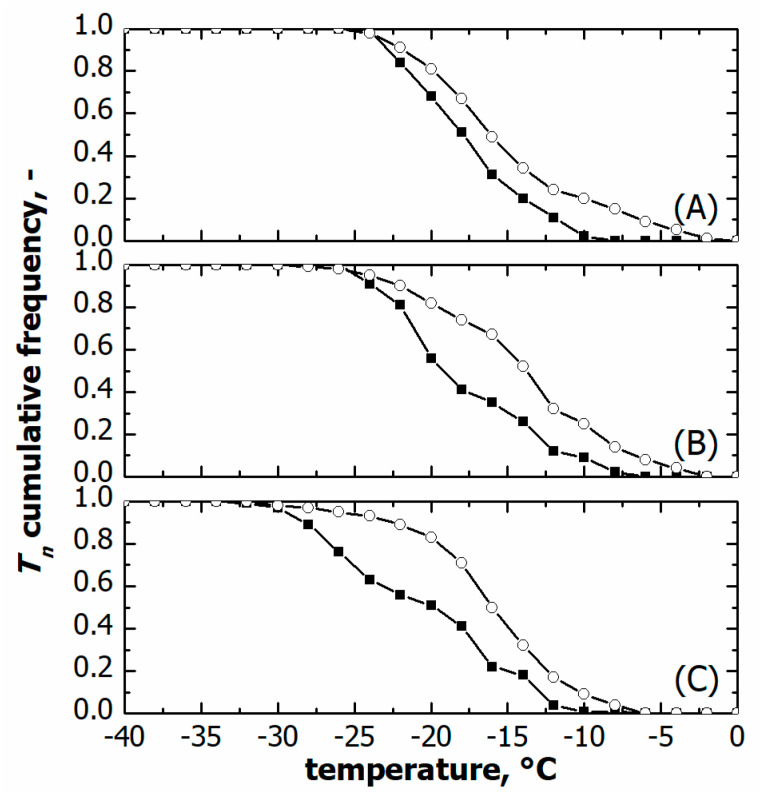
Comparison between cumulative ice nucleation temperature distributions of vials nested in a rack system (○) and in direct contact with the shelf (■). Data refer to various cooling rates: (**A**) 0.25, (**B**) 0.50, and (**C**) 1.00 °C min^−1^.

As shown in Figure 7, vials nested in a rack system tended to nucleate at higher temperatures, i.e., both *T*_10_ and *T*_50_ were higher compared to vials directly resting on the shelf, see Table 1. *T*_90_ did not vary at moderate cooling rates, 0.25 and 0.5 °C min^−1^, while it was significantly lower in the case of vials resting on the shelf and processed at 1 °C min^−1^. These different ice nucleation temperature distributions certainly impacted the average size distribution of ice crystals and, hence, the lyophilised cake pores.

**Figure 8:**
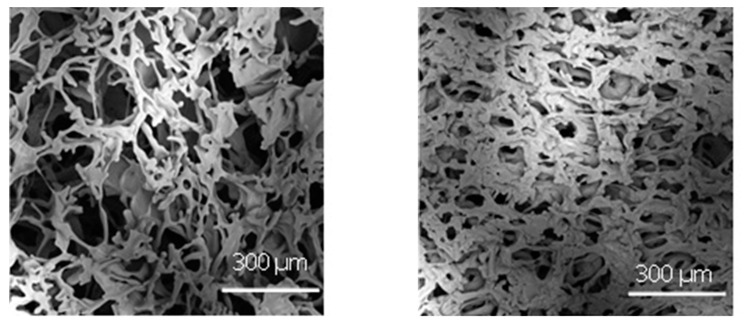
SEM micrographs at the dried cake centre in the case of (**left**) vials nested in a rack system and (**right**) in direct contact with the shelf. Freezing was conducted using a cooling rate of 0.5 °C min^−1^.

Figure 8 compares the porous structure of 5% sucrose in the case of vials nested in a rack system and those directly resting on the shelf. The former exhibited larger pores in agreement with the higher nucleation temperature shown in Figure 7. More specifically, the average pore size of the sample in vials nested in a rack system was 170 µm versus 75 µm observed for the vials directly resting on the shelf. Because of the amorphous nature of the lyophilised samples, there should be a direct relationship between the average pore size and the specific surface area. The specific surface area of the lyophilised samples confirmed this behaviour. In the case of vials nested in a rack system, the specific surface area was significantly smaller than that observed for the vials in direct contact with the shelf, 0.450 ± 0.010 m^2^g^−1^ versus 0.610 ± 0.012 m^2^g^−1^. Consequently, based on the product morphology, we expect that most vials nested in a rack system show shorter primary drying and more prolonged secondary drying at a constant temperature than vials resting on the shelf.

The two loading configurations were thus used to freeze–thaw an enzyme, i.e., lactate dehydrogenase, which is sensitive to the ice–water interface [15,17]. The LDH residual biological activity after two freeze/thawing runs at 0.25 °C min^−1^ was 36.9% for vials nested in a rack system versus 17.1% for vials resting on the shelf. The *p*-value was lower than 0.05; thus, there is sufficient statistical evidence to conclude that the two loading configurations differed in the LDH bioactivity recovery. Furthermore, these results agree with the specific surface area measurements; the vials resting on the shelf showed a more extensive specific surface area and, thus, were more prone to LDH denaturation. Similarly, in the case of freezing at 1 °C min^−1^, the vials nested in a rack system better preserved the LDH bioactivity than those directly resting on the shelf, 33.9% versus 21.8%. On the contrary, the cooling rate did not impact the LDH bioactivity recovery on constant loading configurations.

## 4. Conclusions

This study has compared the freezing behaviour of vials directly resting on the shelf and nested in a rack system. In the case of a rack system, the vials tended to nucleate at higher temperatures, promoting the formation of larger ice crystals and, thus, larger pores in the case of lyophilisation. This result is undoubtedly beneficial to all those active molecules prone to denaturation because of interactions with the ice–water interface. Furthermore, the loading configuration impacts the product morphology after lyophilisation but also its behaviour during drying. Consequently, if a cycle is developed on a laboratory-scale freeze-dryer using a rack system, its scale-up in a production unit should account for the different freezing behaviour as it affects the product morphology and, thus, its resistance to vapour flow during primary drying. Therefore, it is recommended to continue these studies further to assess at a larger scale if the differences seen at the laboratory scale can positively impact the overall lyophilisation cycle process and the drug product stability.

## Figures and Tables

**Table 1:** Percentile values of the cumulative nucleation time and temperature distributions.

Loading Config.	Cooling Rate, °C min^−1^	*t*_10_, min	*t*_50_, min	*t*_90_, min	(*t*_90_-*t*_10_), min	*t*_IQR_, min	*T*_10_, °C	*T*_50_, °C	*T*_90_, °C	(*T*_90_-*T*_10_), °C	*T*_IQR_, °C
Direct	0.25	50	87	107	57	28	−12	−18	−23	−11	−6
Direct	0.50	30	47	57	27	25	−11	−19	−23	−12	−7
Direct	1.00	15	25	36	21	24	−13	−20	−29	−16	−9
Nested	0.25	45	86	110	65	37	−6	−16	−22	−16	−6
Nested	0.50	23	40	65	42	15	−7	−14	−22	−15	−8
Nested	1.00	18	22	34	16	6	−10	−16	−22	−12	−6

## Data Availability

Data are available upon request to the corresponding author.

## Abbreviations

A	cross-sectional area of the vial, m^2^
${\hat{c}}_{p,\ell }$	specific heat capacity of the liquid being frozen, J kg^−1^K^−1^
$F_{n}$	cumulative fraction of nucleated vials, –
$j_{q}$	heat flow rate, W
*N*_j_	number of vials of *j*-class, –
$N_{t}$	total number of monitored vials, –
P	pressure, Pa
$P_{0}$	saturation pressure, Pa
t	time, s
$t_{10}$	10th percentile of the nucleation time data, min
$t_{50}$	median nucleation time, min
$t_{90}$	90th percentile of the nucleation time data, min
$t_{n}$	ice nucleation time, min
$T_{10}$	10th percentile of the nucleation temperature data, °C
$T_{50}$	median nucleation temperature, °C
$T_{90}$	90th percentile of the nucleation temperature data, °C
$T_{\ell }$	temperature of the liquid being frozen, °C
$T_{n}$	ice nucleation temperature, °C
$T_{s}$	shelf temperature, °C
U	overall heat transfer coefficient, Wm^−2^K^−1^
$V_{\ell }$	volume of the liquid being frozen, m^−3^
Greek letters	
$\rho_{\ell }$	mass density of the liquid being frozen, kg m^−3^
Abbreviations	
PVDF	polyvinylidene fluoride
*t*_IQR_	interquartile range of the nucleation time distribution, min
*T*_IQR_	interquartile range of the nucleation temperature distribution, °C
